# Design, Analysis and Simulation of a MEMS-Based Gyroscope with Differential Tunneling Magnetoresistance Sensing Structure

**DOI:** 10.3390/s20174919

**Published:** 2020-08-31

**Authors:** Cheng Li, Bo Yang, Xin Guo, Xinru Chen

**Affiliations:** 1 School of Instrument Science and Engineering, Southeast University, Nanjing 210096, China; 230198302@seu.edu.cn (C.L.); 230179247@seu.edu.cn (X.G.); 220193320@seu.edu.cn (X.C.); 2 Key Laboratory of Micro-Inertial Instruments and Advanced Navigation Technology, Ministry of Education, Nanjing 210096, China

**Keywords:** MEMS gyroscope, tunneling magnetoresistance, planar multi-turn micro-coil, differential detection

## Abstract

The design, analysis, and simulation of a new Micro-electromechanical System (MEMS) gyroscope based on differential tunneling magnetoresistance sensing are presented in this paper. The device is driven by electrostatic force, whereas the Coriolis displacements are transferred to intensity variations of magnetic fields, further detected by the Tunneling Magnetoresistance units. The magnetic fields are generated by a pair of two-layer planar multi-turn copper coils that are coated on the backs of the inner masses. Together with the dual-mass structure of proposed tuning fork gyroscope, a two-stage differential detection is formed, thereby enabling rejection of mechanical and magnetic common-mode errors concurrently. The overall conception is described followed by detailed analyses of proposed micro-gyroscope and rectangle coil. Subsequently, the FEM simulations are implemented to determine the mechanical and magnetic characteristics of the device separately. The results demonstrate that the micro-gyroscope has a mechanical sensitivity of 1.754 nm/°/s, and the micro-coil has a maximum sensitivity of 41.38 mOe/µm. When the detection height of Tunneling Magnetoresistance unit is set as 60 µm, the proposed device exhibits a voltage-angular velocity sensitivity of 0.131 mV/°/s with a noise floor of 7.713 × 10^−6^°/s/$\sqrt{\mathrm{Hz}}$ in the absence of any external amplification.

## 1. Introduction

Through decades of development, the Micro-electromechanical System (MEMS) gyroscopes for rotation sensing have grown into an indispensable component of civil and military applications, such as consumer electronics, industrial robots, automobiles, and navigation systems [1,2]. For the purpose of gradually replacing the strategic gyroscopes with undesired large volume and expensive cost, the demand for better performances of current MEMS gyroscopes is exquisitely increasing [3]. Therefore, much attention has been paid and then several approaches have been proposed, including digitization technology, temperature compensation method, mode-matching technique, force rebalance control, etc. [4,5,6,7]. Nevertheless, the bias stability of capacitive micro-gyroscopes is rather hard to improve to rival with optical gyroscopes due to the influences of parasitic capacitance effect and noise in the sense interface [8]. In light of this, exploring an alternative MEMS-based sensing strategy with greater potential is full of significance.

The quantum tunneling effect, which arises from lower-energy particles passing through the higher-energy barrier, exhibited a bright prospect [9]. Owing to the prominent dexterity of tunneling current, several micromachined tunneling gyroscopes were designed and reported. According to the literatures [10,11,12,13], the majority of this type devices utilize the drastic variation of tunneling current between tunneling tip and metal electrode in order to detect the angular velocity. Unfortunately, the tunneling effect is only existent in the condition that the gap is no more than approximate 1 nm, which will invalidate the device when it undergoes a slightly large Coriolis force [12,13]. In other words, the dynamic ranges of these devices are extremely narrow. Simultaneously, it is difficult to precisely control the distance of 1 nm under current micromachining fabrication technology [14]. Thus, the development of traditional tunneling gyroscopes is in great limitation.

Tunneling Magnetoresistance (TMR) effect at present is another effective measurement technique that is widespread in the detection of structural deformation caused by pressure, displacement, and inertial quantities, etc. [15,16,17,18]. The tunneling current of TMR sensor is governed by the changes in direction and intensity of the external magnetic field, which permits a broader dynamic interval than quantum tunneling effect [17,18]. The first TMR-based pressure sensor was directly made from Magnetic Tunnel Junctions (MTJs) [15], which utilized the magnetization rotation to change the TMR value, further detecting the external strain or stress. The fabricated prototype realized the linear measurement with an unsurpassed gauge factor of up to 840. The subsequent improved design of this device upgraded the sense layer with CoFeB material [19]. The experiments revealed that the improved one could successfully differentiate between compressive and tensile stress. Another MTJs-based MEMS pressure sensor was reported in [20] and it has the same CoFeB/MgO/CoFeB structure, as above. The TMR value of this device was much more than 200% for polycrystalline sense layers and the gauge factor raised to 3406 in a range of 600 mbar pressure. Literature [16] presented a displacement sensor comprised of a deposited magnetoresistance (MR) sensor and a movable microstructure bonding with a magnetic film. The local magnetic field of the MR sensor concerned with the deformation of microstructure. Hence, the displacement of microstructure could be acquired through demodulating the output of MR sensor. Literatures [17,21] proposed a TMR-based MEMS accelerometer using three-dimensional (3D)-print and micromachined sensing structures respectively. Both of them adopted an integrated TMR sensor to detect the position change of the permanent magnet, further recognizing the input acceleration. The experimental results indicated that the final sensitivities of the two devices are 409.01 mV/g and 8.85mV/g, respectively. Literature [22] reported another accelerometer with the similar operating principle to ones from [17,21]. The peculiarity of latter one is the use of the simpler but more effective micro-cantilever sensing structure, and the test results demonstrated the system could achieve measurement resolution of 17.35 µg/$\sqrt{\mathrm{Hz}}$. TMR-based gyroscopes having been reported is quite rare until now. Literature [23,24,25] developed a linear TMR angle transducer and its signal conditioning circuit in detail. In absence of the Coriolis principle, this transducer directly employed a permanent magnet for the rotation target and utilized the TMR sensing unit to sense revolving magnetic field. The latest record shows that the output of prototype varies linearly over 360° range with a worst-case non-linearity of less than 0.53%. A new TMR micromachined gyroscope featuring low noise was designed in [18], whose principle resembles to the traditional Coriolis micro-gyroscope. The device adopted a pair of magnets in driving axis to provide magnetic driving force and simultaneously arranged a magnet in sensing axis to yield variating magnetic field. The magnetic field measurement is implemented by a TMR sensor and the theoretical sensitivity is 1.18 mV/°/s with a noise floor of 9.48 × 10^−5^°/s/$\sqrt{\mathrm{Hz}}$.

In summary, the constructions of existing TMR-based sensors involve the design of TMR structure and force-deformation conversion mechanism, as well as how to incorporate them to a whole. When it comes to inertial devices, most of above accelerometers and gyroscopes adopt magnetic film or permanent magnet to generate magnetic field; however, it is not conducive to the high integration and mass fabrication. Another important issue having not been noticed is the suppression of external electromagnetic interference. Especially in gyroscopes, the Coriolis displacement of which is so week that is easily overwhelmed by external noise and crosstalk from drive mode. Therefore, technologies, such as micro-coil and differential detection, could be taken into account for improving the integration and performances of current designs.

In this work, we develop a novel MEMS-based TMR gyroscope with differential sensing structure. We utilize comb structure to electrostatically drive proof mass whilst adopting two double-layer rectangle coils to generate reversely variating magnetic field in sensing axis. Two pairs of differential TMR sensing units are respectively arranged under two micro-coils in order to construct a two-stage differential detection. The device design and analysis are presented in detail in Section 2; Section 3 demonstrates the simulation results and feasibility analysis. The conclusion and concentration of future work are finally given Section 4.

## 2. Device Design

### 2.1. Overall Conception

**Figure 1:**
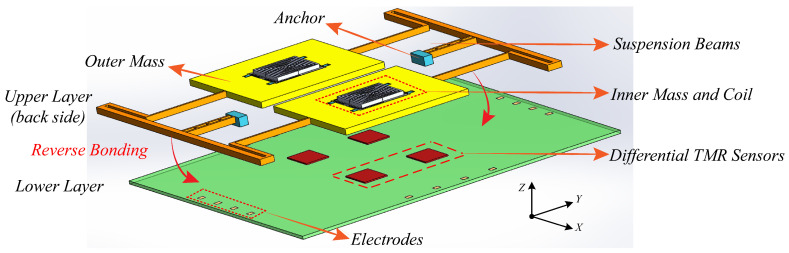
The schematic layout of proposed TMR-based MEMS gyroscope.

The schematic diagram of TMR-based micro-gyroscope that is illustrated in Figure 1 is composed of upper mechanical structure and lower substrate layer. The upper layer of mechanical sensing structure is a typical tuning fork gyroscope (TFG), which allows for the differential detection of angular velocity due to the dual-mass design [26]. The uniqueness of the proposed scheme is plating a two-layer planar micro-coil on the emptied inner mass to generate a constant magnetic field when the copper wire is applied with an exciting current. When compared with the permanent magnet used in previous works, the magnetic field yielded by energizing coil can avoid demagnetization and, thereby, possesses a better long-term stability [27]. Simultaneously, the intensity of magnetic field can be adjusted via current value, which can effectively alleviate the problem of magnetic saturation. The pair of outer masses are driven to vibrate in reverse directions along X axis, so that the pair of inner masses move reversely along Y axis when an angular velocity is inputted. Thus, the pair of copper micro-coils form two independent magnetic fields with differential variation in sense axis.

**Figure 2:**
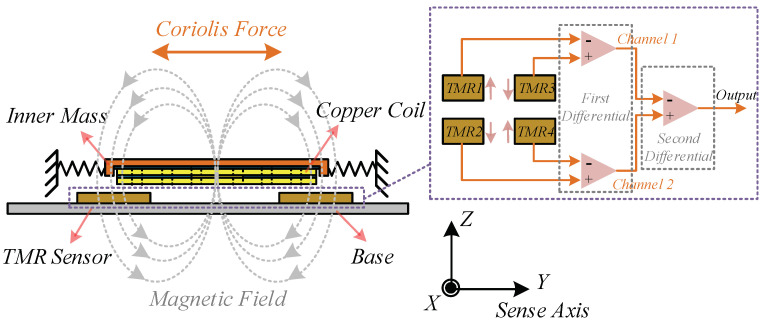
The lateral sketch of the micro-gyroscope.

A pair of Y-sensed TMR units are symmetrically arranged underneath each coil to differentially detect the local variations of magnetic fields, owing to the symmetrical distribution of magnetic field along Y axis, as shown in Figure 2. The dual-mass design of TFG and symmetrical layout of TMR sensors construct a two-stage differential measurement. Therefore, the mechanical common-mode errors stemming from Y-axis acceleration, and TMR common-mode errors caused by electromagnetic interference can be restricted concurrently. Moreover, the device sensitivity is improved by four times and the Signal to Noise Ratio (SNR) is refined effectively. In addition, it is noteworthy that the positions of TMR sensors are set to the points with maximum variation rate of magnetic field intensity to achieve the optimum measuring sensitivity.

### 2.2. Mechanical Structure

**Figure 3:**
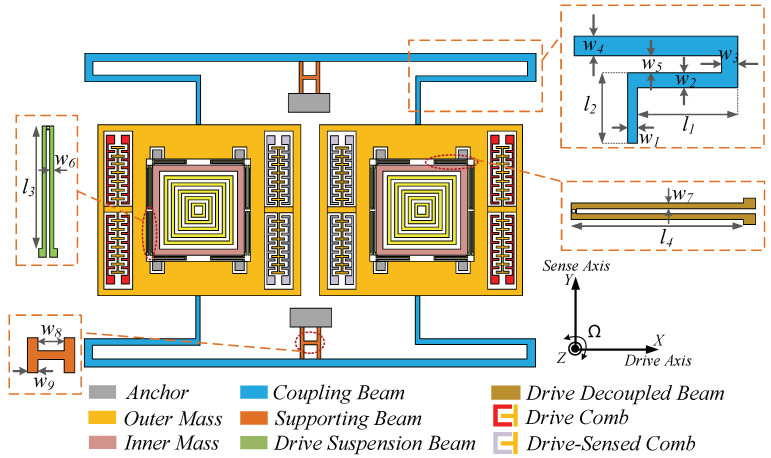
The structure of dual-mass micro-gyroscope.

The dual-mass TFG is comprised of two symmetrically arranged substructures, as illustrated in Figure 3. Two identical submodules are connected by two T-shape suspensions, coupling them to vibrate in the same frequency along both driving and sensing axes (X-axis and Y-axis). The T-shape beam further consists of a fishhook-shape and ladder-shape architectures. The fishhook-shape structure ensures enough flexibility in both driving and sensing axes, while the ladder-shape architecture effectively isolates the in-phase and anti-phase movements of drive and sense modes concurrently [28,29]. Each substructure of the TFG contains an outer mass serving as drive module and an inner mass that responds to Coriolis force. Two masses are connected by the drive beams which are longer than drive-decoupled beams to balance the stiffness between drive and sense directions. The width and length (*w*_4_ and *l*_5_) of upper straight suspension of the coupling beam are designed carefully to ensure enough coupling stiffness in sense mode whereas the width of connection end (*w*_1_) is optimized in ordr to acquire appropriate coupling stiffness in drive direction. Table 1 gives the concrete parameters of the proposed structure.

**Figure 4:**
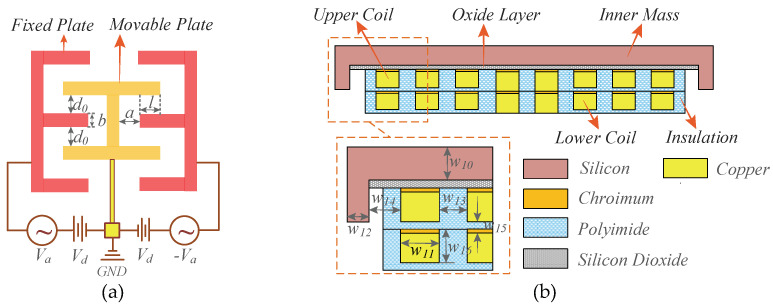
Locally enlarged view of the micro-gyroscope: (**a**) The drive comb; and (**b**) The copper coil.

A pair of drive combs and drive-sensed combs are respectively set on the outer and inner edges of the outer masses to implement the closed-up driving. The bilateral fixed plates of drive combs are applied with reverse drive voltages of *V_d_* + *V_d_*$\sin\left(w_{d}t\right)$ and *V_d_* − *V_d_*$\sin\left(w_{d}t\right)$ to form “push-pull” driving, as shown in Figure 4a. Because of the large stiffness of four U-shaped drive decoupled beams in drive axis, the inner mass is incapable of vibrating with outer mass in drive axis, thereby suppressing the cross-talk of magnetic field from drive axis. When an external angular velocity is exerted in Z axis, four drive suspensions force two proof masses to move in sense axis together, further generating the variations of magnetic fields. The two-layer multi-turn copper coils are electroplated on the grooves of the back sides of the inner masses to ensure adequate magnetic intensity. Before growing coils, the silicon dioxide layers are coated firstly on the attached end to avoid the electrical conduction between coil and masses. Moreover, to enhance the adhesion between silicon dioxide and copper, the connection layers of chromium are sputtered in advance serving as the bases, which is depicted in Figure 4b. In particular, the polyimide layers are exploited in order to cover the upper coils and construct new planes for the forming of lower coils [30].

**Figure 5:**
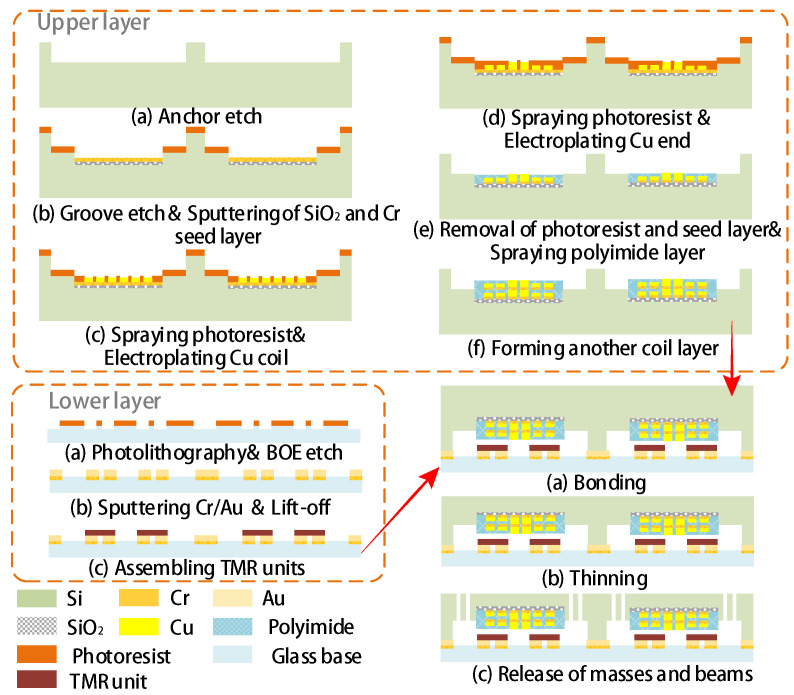
The simplified fabrication process of proposed device.

Figure 5 illustrates the simplified fabrication process. The upper mechanical structure begins with a deep etch of anchors and the subsequent etch of grooves, within which the copper coils will be coated. Without removing the residual photoresist, a layer of SiO_2_ for insulation and Cr as seed are sputtered successively. Subsequently, the upper coil is electroplated after another spraying of photoresist and photolithography. To link the two layers of coil, the Cu connecting ends are electroplated subsequently and after which, the residual photoresist is striped and the seed layer is etched to avoid the short-circuit between each turn of the coil. Meanwhile, the inlet and outlet of the exciting current are also coated at this stage, which are routed to the surfaces of two inner anchors respectively, further exported by lead wires. Afterwards, the polyimide layers are sprayed and another layer of coil is formed on the new plane by the same steps. The fabrication of lower substrate structure involves the sputtering of electrodes and micro-assembling of the TMR units. Because the positions of TMR units have a great influence on the sensitivity of the device, the micro-cursor alignment marks will be used in both TMR assembling and the subsequent bonding to minimize the alignment errors.

### 2.3. Coil Structure

#### 2.3.1. Magnetic Field Distribution

**Figure 6:**
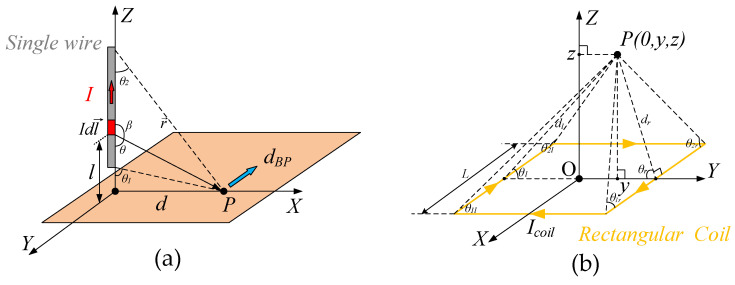
The mathematical model of single wire and single rectangular coil: (**a**) Single wire; and, (**b**) Rectangular coil.

The multi-turn micro-coil adopted generate magnetic field is equivalent to the linear superposition of single-circle rectangle coils based on the vector nature of magnetic field. Due to the symmetry of square architecture, the single loop can be further disintegrated to four identical electrified wires as depicted in Figure 6a. Assuming the exciting current (*I*) conducts along Z axis, the magnetic induction intensity (*MII*) in point P (*B_P_*) produced by the infinitesimal ($Id\vec{l}$) can be derived as [31]:

(1) $$d\vec{B_{P}}=\frac{\mu_{0}}{4\pi }\frac{Id\vec{l}\times \vec{r}}{r^{3}}$$

where $\mu_{0}$ is vacuum permeability; $\vec{r}$ is the vector distance between current infinitesimal and point P, whereas *r* is the scalar distance. Since magnetic fields induced by different segments of the wire have the same direction (Y-Axis), the resultant *MII* in point P can be integrated as:

(2) $$B_{P}=\frac{\mu_{0}I}{4\pi }\int_{L}\frac{dl\sin\beta }{r^{2}}$$

Considering:

(3) $$\left\{ \begin{array}{c} \beta =\pi -\theta \\ l=d\cot\theta \\ r=\csc\theta \end{array}\right.$$

where *d* is the distance between point P and origin, Equation (2) can be further converted to integral, which takes θ as independent variable and the final result is:

(4) $$B_{P}=\frac{\mu_{0}I}{4\pi d}\left(\cos\theta_{2}-\cos\theta_{1}\right)$$

Figure 6b illustrates the rectangular coil model. We assume the point P locates over the coil plane and vibrates along Y axis. According to the single-wire analysis, the directions of *MIIs* in point P produced by the front and rear wires are along X axis. However, the TMR sensors we adopt have the unidirectional sensing structure and are arranged along Y axis. Besides, the front and rear magnetic fields are ideally counteracted in X axis due to the opposite currents. Therefore, the operations of TMR sensors are barely influenced by the front and rear wires. In fact, mere the right and left wires are effective constituents in our design. Based on the Equation (5) and parameters in Figure 6b, the *MIIs* in point P yielded by right and left wires can be respectively expressed as:

(5) $$\left\{ \begin{array}{c} B_{r}=\frac{\mu_{0}I_{coil}}{4\pi d_{r}}\left(\cos\theta_{r2}-\cos\theta_{r1}\right)\\ B_{l}=\frac{\mu_{0}I_{coil}}{4\pi d_{l}}\left(\cos\theta_{l2}-\cos\theta_{l1}\right) \end{array}\right.$$

where

(6) $$\left\{ \begin{array}{c} d_{r}=\sqrt{{\left(\frac{L}{2}-y\right)}^{2}+z^{2}}\\ d_{l}=\sqrt{{\left(\frac{L}{2}+y\right)}^{2}+z^{2}} \end{array}\right.$$

(7) $$\left\{ \begin{array}{c} \cos\theta_{r2}=-\cos\theta_{r1}=\frac{L}{\sqrt{L^{2}+4{d_{r}}^{2}}}\\ \cos\theta_{l2}=-\cos\theta_{l1}=\frac{L}{\sqrt{L^{2}+4{d_{l}}^{2}}} \end{array}\right.$$

and $I_{coil}$ is the exciting current of the coil; *L* is the length of single wire; *d*_*r*(*l*)_ is the distance between point P and right wire(left) wire; $\theta_{r(l)1}$ and $\theta_{r(l)2}$ are the included angles between point P and two ends of right (left) wire, respectively; and, *y* and *z* are the abscissa and ordinate of point P. According to Biot–Savart Law, the directions of right and left magnetic fields are perpendicular to the two planes determined by right wire versus point P, and left wire versus point P, concurrently. Thus, we utilize orthogonal decomposition to acquire the Y-axis components of right and left magnetic fields, which are:

(8) $$\left\{ \begin{array}{c} B_{ry}=B_{r}\sin\theta_{r}\\ B_{ly}=B_{l}\sin\theta_{l} \end{array}\right.$$

where $\sin\theta_{r(l)}$ is the included angle between point P and coil plane, and

(9) $$\left\{ \begin{array}{c} \sin\theta_{r}=\frac{z}{d_{r}}\\ \sin\theta_{l}=\frac{z}{d_{l}} \end{array}\right.$$

According to the Ampere rule, $B_{ry}$ and $B_{ly}$ have the reverse directions, therefore, the resultant *MII* of single coil in point P along X axis is

(10) $$B_{P}=B_{ry}-B_{ly}$$

**Figure 7:**
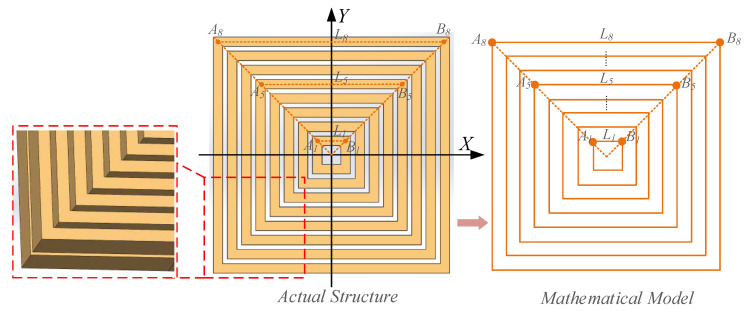
The model of proposed multi-turn rectangular micro-coil.

Because the proposed multi-turn coil shown in Figure 7 consists of single loops with diverse dimensions, the total *MII* of multi-turn coil can be given as:

(11) $$B_{P\Sigma }=\sum_{i=1}^{8}B_{P_{i}}$$

where $B_{P_{i}}$ denotes the *MII* of *i*-th turn coil. The concrete lengths of each circle are shown in Table 2.

**Figure 8:**
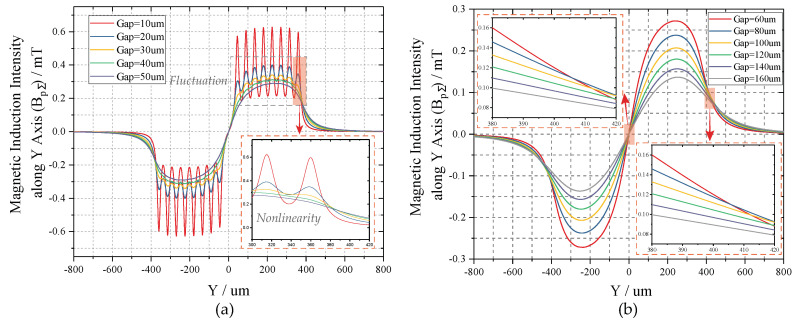
The magnetic field distributions along Y axis under different heights: (**a**) z < 60 µm; and, (**b**) z ≥ 60 µm.

Because the cross-sectional area of the single wire is 60 µm × 30 µm, that of the designed two-layer coil can be equivalent to 120 µm × 30 µm in calculation. The safety current of copper conductor is generally no more than 10A per square millimeter. Thus, we take the criteria of 8A per square millimeter to set the exciting current $I_{coil}$ (approx. 28 mA) in simultaneous consideration of the desired magnetic intensity. To analyze the magnetic field distribution along Y axis, the variation interval of *y* is set from −800 µm to 800 µm to cover the whole coil. When considering that the detection height has a great influence on magnetic intensity, the gap between coil plane and TMR sensors is set to increase from 10 µm to 50 µm with a step of 10 µm, and from 60 µm to 160 µm with a step of 20 µm, separately. Figure 8 illustrates the magnetic field distributions of proposed copper coil. In Figure 8a, the magnetic field curves exhibit great fluctuations around the peak and valley where the gap is smaller than 60 µm, which will lead to a severe nonlinearity in magnetic variation. Consequently, despite possessing larger magnetic intensity, gaps less than 60 µm are not suitable for linear measurement in this design. Differently, the magnetic strengths under locations higher than 60 µm present much smoother variating process, as indicated in Figure 8b. The whole magnetic field has an odd symmetric distribution about origin, facilitating the differential detection. The maximum of *MII* decreases from 0.272 mT to 0.137 mT with the gap extends from 60 µm to 160 µm. From the inset, we can find that there is a wide linear region located on both sides of the peaks; therefore, we take the gap larger than 60 µm as the operating circumstance.

In particular, it is noteworthy that the number of the coil turns and the driving current directly decide the performance of the micro-coil. However, the magnetic intensity as well as its sensitivity is linearly proportional to the driving current which is demonstrated in Equation (5) and (12). Thus, increasing the exciting current brings greater benefits to the coil performances than the number of turns. To this end, we give prior consideration to the expansion of the cross-sectional area of single-loop coil. Nevertheless, with the reduce of the turns, the fluctuation around the peak and valley will remain until a higher gap, which leads to a severer loss of magnetic intensity. Therefore, we design the micro-coil with a turns number of eight and a cross-sectional area of 60 µm × 30 µm.

#### 2.3.2. Sensitivity Analysis

Sensitivity analysis of magnetic field variation is implemented to determine the optimal detection points in order to coordinate the measurement accuracy and linear range of TMR sensors. Because condition of gap < 60 µm is not suitable for linear measurement, only gaps greater than 60 µm are taken into account. We take y as independent variable, and the partial differential of Equation (8) can be deduced as:

(12) $$\left\{ \begin{array}{c} \frac{\partial B_{ry}}{\partial y}=-\frac{4\mu_{0}zI_{coil}}{\pi }\cdot \frac{L_{ry}\left(3{L_{ry}}^{2}+12z^{2}+2L^{2}\right)}{{\left({L_{ry}}^{2}+4z^{2}\right)}^{2}\cdot {\left({L_{ry}}^{2}+4z^{2}+L^{2}\right)}^{\frac{3}{2}}}\\ \frac{\partial B_{ly}}{\partial y}=-\frac{4\mu_{0}zI_{coil}}{\pi }\cdot \frac{L_{ly}\left(3{L_{ly}}^{2}+12z^{2}+2L^{2}\right)}{{\left({L_{ly}}^{2}+4z^{2}\right)}^{2}\cdot {\left({L_{ly}}^{2}+4z^{2}+L^{2}\right)}^{\frac{3}{2}}} \end{array}\right.$$

where

(13) $$\left\{ \begin{array}{c} L_{ry}=L-2y\\ L_{ly}=L+2y \end{array}\right.$$

Hence, the partial differential of total *MII* of multi-turn coil can be expressed as

(14) $$S_{B_{p\Sigma }}=\frac{\partial B_{p\Sigma }}{\partial y}=\Sigma \left(\frac{\partial B_{ry}}{\partial y}-\frac{\partial B_{ly}}{\partial y}\right)$$

**Figure 9:**
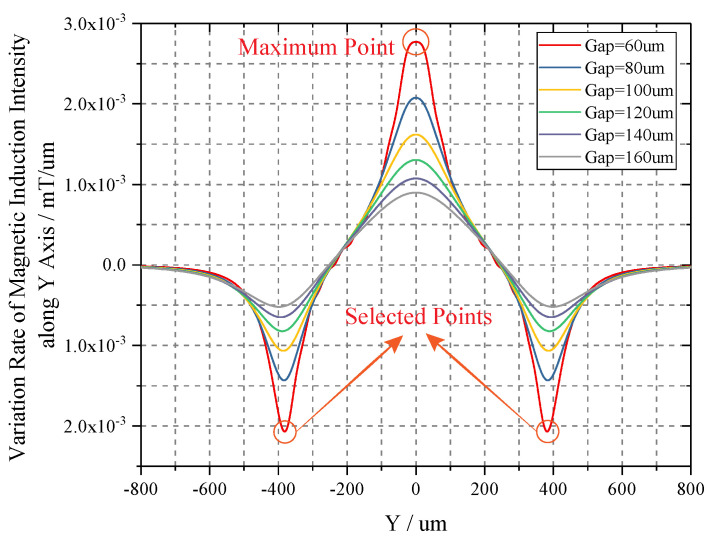
Sensitivity curves of magnetic induction intensity along Y axis.

Here, we plot the sensitivity curves of *MII* along Y axis in Figure 9. It can be found that the maximum sensitivity emerges at the points of y = 0 µm, i.e., the geometric center of the multi-turn coil. Additionally, there are another pair of two symmetrical extremums situated on the two sides of the maximum, respectively. Although all of the three candidates are enclosed in regions with great linearity, in order to realize two-stage differential detection, we select the bilateral extremums as the final sensing points. Meanwhile, with the reduce of detection height, the sensitivity increases monotonously, which is in line with *MII*.

The extremums of sensitivity curves are further analyzed in order to acquire the specific sensitivity values. Taking y as independent variables, the partial differential of Equation (12) can be derived as:

(15) $$\left\{ \begin{array}{c} \frac{\partial (\frac{\partial B_{ry}}{\partial y})}{\partial y}=-\frac{4\mu_{0}zI_{coil}}{\pi }\cdot \frac{\left({L_{ry}}^{2}+m\right){\left({L_{ry}}^{2}+q\right)}^{\frac{1}{2}}\left[9\left({L_{ry}}^{2}+m\right)\left({L_{ry}}^{2}+n\right)\left({L_{ry}}^{2}+q\right)-{L_{ry}}^{2}\left(3{L_{ry}}^{2}+m\right)\left(7{L_{ry}}^{2}+3m+4q\right)\right]}{{\left({L_{ry}}^{2}+n\right)}^{4}{\left({L_{ry}}^{2}+q\right)}^{2}}\\ \frac{\partial (\frac{\partial B_{ly}}{\partial y})}{\partial y}=-\frac{4\mu_{0}zI_{coil}}{\pi }\cdot \frac{\left({L_{ly}}^{2}+m\right){\left({L_{ly}}^{2}+q\right)}^{\frac{1}{2}}\left[9\left({L_{ly}}^{2}+m\right)\left({L_{ly}}^{2}+n\right)\left({L_{ly}}^{2}+q\right)-{L_{ly}}^{2}\left(3{L_{ly}}^{2}+m\right)\left(7{L_{ly}}^{2}+3m+4q\right)\right]}{{\left({L_{ly}}^{2}+n\right)}^{4}{\left({L_{ly}}^{2}+q\right)}^{2}} \end{array}\right.$$

where

(16) $$\left\{ \begin{array}{c} n=4z^{2}\\ q=4z^{2}+L^{2}\\ m=n+2q \end{array}\right.$$

Afterwards, we get:

(17) $$\frac{\partial S_{B_{p\Sigma }}}{\partial y}=\Sigma \left(\frac{\partial (\frac{\partial B_{ry}}{\partial y})}{\partial y}-\frac{\partial (\frac{\partial B_{ly}}{\partial y})}{\partial y}\right)$$

**Figure 10:**
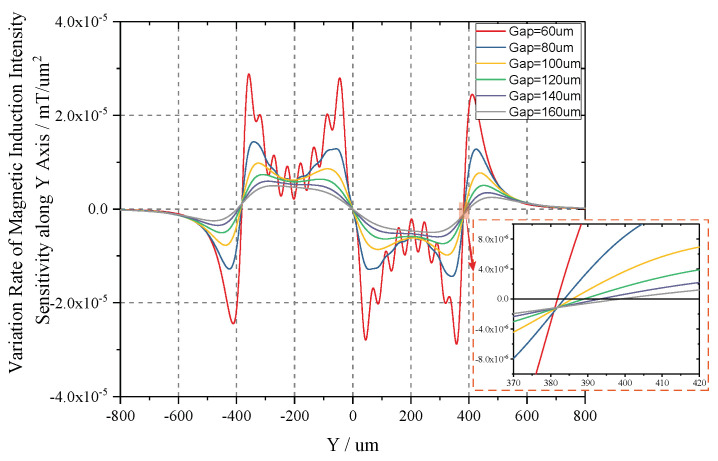
First derivative curve of the sensitivity of magnetic induction intensity along Y axis.

The curves of Equation (17) are illustrated in Figure 10, and we solve the equation of $\frac{\partial S_{B_{p\Sigma }}}{\partial y}$ = 0. The corresponding extremums are obtained in Table 3. When the gap equals 60 µm, the variating rate of *MII* along Y axis is 0.002069 mT/µm, and the corresponding magnetic field intensity of 20.69 mOe/µm. The usage of differential detection can improve the sensitivity (*S*_*m*−*d*_) by two time, which is 41.38 mOe/µm. In comparison with choosing maximum point with a sensitivity of 27.7 mOe/µm, the differential detection enhances the sensitivity by 1.49 times, and enables the rejection of the common-mode errors causing by TMR sensors concurrently. Meanwhile, the magnetic field intensity in the selected points (Gap = 60 µm) are ±1.24 Oe. Thus, the voltage-displacement sensitivity (*S*_*v*−*d*_) can be calculated by:

(18) $$S_{v-d}=S_{v-d}\times S_{m-d}$$

where *S*_*v*−*m*_ is the voltage-MII sensitivity that is determined by the TMR sensor. The TMR sensor we adopted is TMR 9001 provided by Multi Dimension Technology Corporation with an ultra-high sensitivity (*S*_*v*−*m*_) of 300mV/V/Oe and a linear range of −2∼2 Oe [21,32]. Therefore, the differential sensors can linearly operate in the magnetic field generated by proposed multi-turn coil with a voltage-displacement sensitivity of 12.414 mV/V/µm.

## 3. Simulation Analysis

### 3.1. Mechanical Simulation

**Figure 11:**
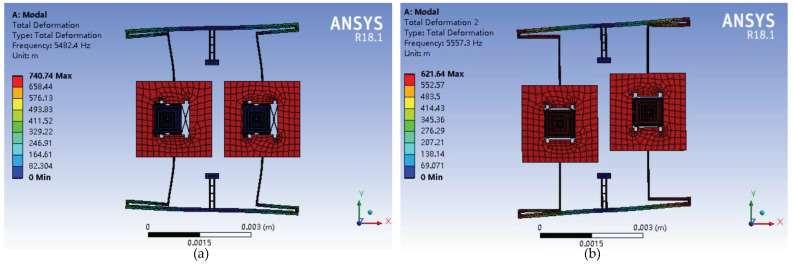

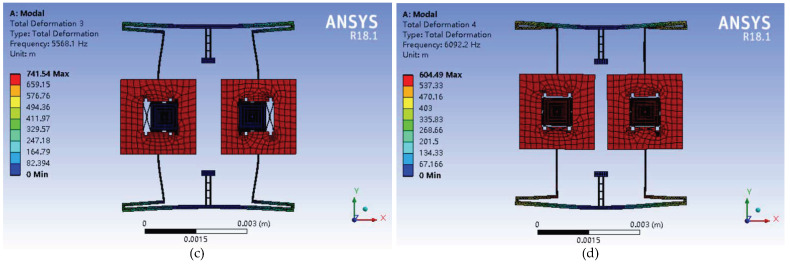
The first four modes of proposed micro-gyroscope: (**a**) The first mode; (**b**) The second mode; (**c**) The third mode; and (**d**) The fourth mode.

Finite Element Method (FEM) simulations are implemented by ANSYS in order to verify the functionality of proposed TFG. The comb structures are translated to equivalent mass of outer proof mass. Structural dimensions are consistent with parameters in Table 1. Modal analysis is carried out firstly to determine the dynamic modes. The first four modes are extracted in Figure 11 and the corresponding inherent frequencies are given in Table 4. It can be found that the first and third modes represent the in-phase and anti-phase movements of drive mode, respectively, meanwhile the second and fourth modes are the anti-phase and in-phase movements of sense mode respectively. Obviously, the second and third modes are the operating modes of dual-mass micro-gyroscope, which are optimized to have a frequency split of 10.8 Hz, ensuring sufficient energy transfer efficiency and working bandwidth. Furthermore, in order to restrict the mode crosstalk, the frequency differences between anti-phase and in-phase vibrations of drive mode is designed as 85.7 Hz and that of sense mode is 534.9 Hz, respectively.

**Figure 12:**
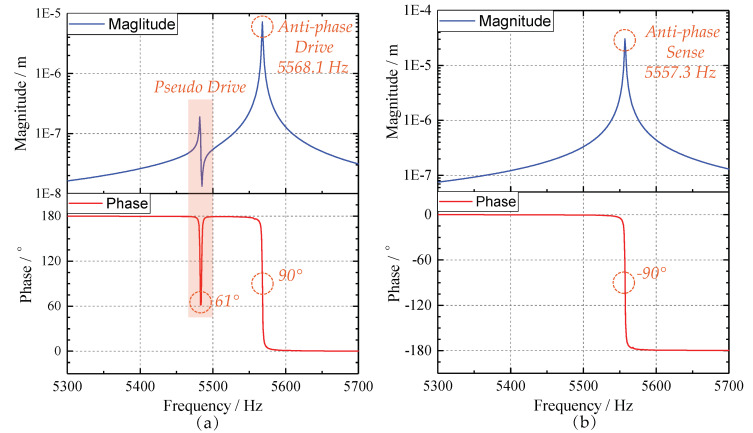
The frequency responses of the proposed micro-gyroscope: (**a**) The drive-mode responses; and (**b**)The sense-mode responses.

The harmonic responses analyses are subsequently performed to determine the frequency response characteristics. We set the damping ratio as 0.0001 and sweep the frequency from 5300 Hz to 5700 Hz. The amplitude-frequency and phase-frequency curves of drive mode are illustrated in Figure 12a, and that of sense mode are plotted in Figure 12b. In Figure 12a, a pseudo mode arising from in-phase movement locates ahead of the anti-phase drive frequency. While the amplitude of this pseudo mode is forty times smaller than that of anti-phase mode. Besides, the phase value (61°) of pseudo resonance mode does not satisfy the requirement (90°) of closed-up control since we employ phase-locked loop (PLL) drive. Hence, the frequency locking in operating mode can hardly be perturbed by the pseudo response.

Finally, the displacement response simulation is implemented to determine the mechanical sensitivity and linearity of the TFG. The input angular rate is tested from −100°/s to 100°/s, which corresponds to the Coriolis force (*F_c_*) from −0.1754 µN to 0.1754 µN [33].

(19) $$F_{c}=2m_{s}\Omega \times \dot{x}=2m_{s}\Omega A_{d}\omega_{d}\cos\left(\omega_{d}-\phi_{d}\right)$$

where *m_s_* is the sense-mode effective mass; Ω is the input angular velocity; $\omega_{d}$ and $\phi_{d}$ are the drive frequency and phase; and, *A_d_* is the amplitude of drive force that can be acquired from:

(20) $$F_{d}=4n\varepsilon V_{d}V_{a}\sin\left(\omega_{d}\right)=A_{d}\sin\left(\omega_{d}\right)$$

where *n* is the number of drive comb; ε, *h* and *d*_0_ denote the vacuum permittivity, comb thickness and comb gap respectively; and, *V_d_* and *V_a_* are the offset and amplitude of drive voltage illustrated in Figure 4. The design values are given in Table 5.

**Figure 13:**
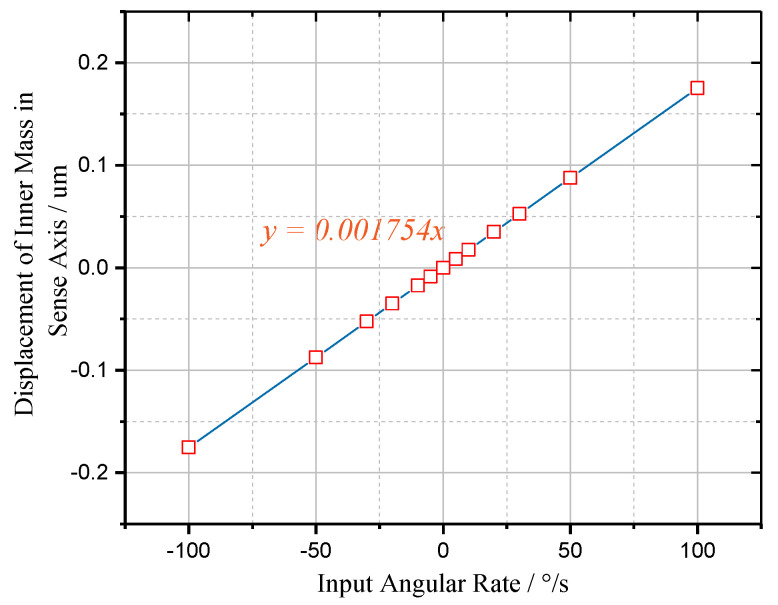
The simulated mechanical sensitivity curve of the proposed micro-gyroscope.

The simulation result presented in Figure 13 demonstrates that the sense mode has a mechanical sensitivity (*S*_Ω_) of 1.754 nm/°/s with an ideal linearity. Incorporating the magnetic sensitivity (*S*_*m*−*d*_) obtained before, the “magnetic intensity − angular velocity” sensitivity (*S*_*m*−Ω_) of single proof mass can be deduced as

(21) $$S_{m-\Omega }=S_{m-d}\times S_{\Omega }$$

Thus, the background noise (*Ni*) of 2.8 PT/$\sqrt{\mathrm{Hz}}$ can be converted to angular velocity noise (*N*_Ω_) as 7.713 × 10^−6^°/s/$\sqrt{\mathrm{Hz}}$ building on Equation (22).

(22) $$N_{\Omega }=\frac{Ni}{S_{m-\Omega }}\times 2$$

(23) $$S_{v-\Omega }=S_{v-d}\times S_{\Omega }\times V_{cc}\times 2$$

Because the two proof masses construct another stage differential detection, the final magnetic sensitivity can be improved by two times. Therefore, according to Equation (23), when the TMR sensor operate in a supply (*V_cc_*) of 3V without any external amplification, the proposed MEMS gyroscope exhibits a “voltage − angular velocity” sensitivity (*S*_*v*−Ω_) of 0.131 mV/°/s.

### 3.2. Magnetic Simulation

**Figure 14:**
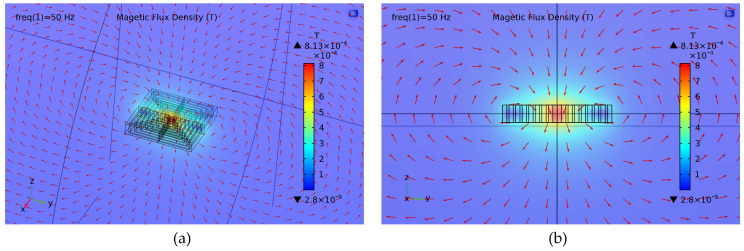
The simulation magnetic field distribution of the proposed multi-turn coil: (**a**) The three-dimensional (3D) perspective; and, (**b**) The Y–Z perspective.

**Figure 15:**
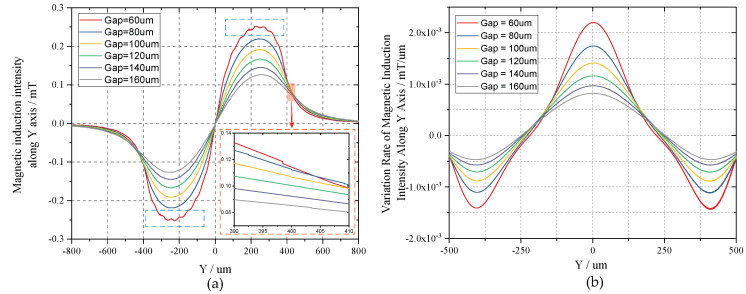
The Y-axis magnetic field distribution of the proposed multi-turn coil: (**a**) The magnetic induction intensity; and, (**b**) The sensitivity curves.

The magnetic properties of proposed multi-turn micro-coil are simulated by COMSOL Multiphysics in this section. Because the distance between two inner mass has exceeded 1500 µm, the cross-talk between the differential coils is so week that can be neglected. Thus, the single coil, whose dimension is in accordance with Table 2, is modelled and simulated. We set the exciting current as 28 mA and the magnetic field distribution is extracted in Figure 14. The red magnetic lines of force indicate the magnetic field directions of the ambient space. From the Y–Z perspective in Figure 14b, we find that the magnetic field has a completely symmetric distribution along Y axis as analyzed in last section. The *MIIs* along Y axis from height of 60 to 160 µm are further extracted in Figure 15a. When compared with the arithmetic results presented in Figure 8, both profile and amplitude are matched, thereby verifying the theoretical formula of Equation (11). Although slight fluctuation has emerged around the peaks in height of 60 µm, the locally enlarged view of Figure 15a demonstrates that the operating interval still has a great linearity. Simultaneously, the sensitivity curves of MIIs are given in Figure 15b. Despite minute error stemming from fitting accuracy, the simulation curves are in great coincidence with theoretical calculation. Therefore, it can be summarized that the devising of this TMR micro-gyroscope are proved viable by above simulations and theoretical analyses.

## 4. Conclusions

We develop a novel MEMS-based TMR gyroscope in this paper. The proposed micro-gyroscope incorporates the high-sensitivity TMR sensing structure with a traditional electrostatic drive technique. Two-stage differential detection is utilized to reject the common-mode errors caused by both mechanical structure and TMR sensors. The integral structure and its operating principle are introduced firstly. Subsequently, to acquire the magnetic characteristics of proposed planar multi-turn coil, the single wire and rectangle coil are modelled and analyzed successively. The analytical results reveal that the proposed coil has a symmetric magnetic field distribution along Coriolis axis and the maximum sensitivity is 41.38 mOe/µm. Subsequently, the mechanical and magnetic simulation are separately implemented by ANSYS and COMSOL Multiphysics. The simulations results demonstrate that when the detection height is 60 µm, the proposed TMR micro-gyroscope exhibits a sensitivity of 0.131 mV/°/s and a noise floor of 7.713 × 10^−6^°/s/$\sqrt{\mathrm{Hz}}$ without any external amplification. Compared with the TMR gyroscope in Literature [18] with a noise floor of 9.48 × 10^−5^°/s/$\sqrt{\mathrm{Hz}}$, that of our design reduced by 12.29 times. Simultaneously, the magnetic film used in Literature [17,21] has a mT-level MII and a mT/mm-level magnetic sensitivity, while the permanent magnet used in Literature [18,22] have a T-level *MII* and an oe/um-level magnetic sensitivity. All of them require a large-range TMR unit to detect the variation of magnetic field such as TMR 2105 and TMR P44FP, or a higher detection gap. However, the TMR sensors with wide range normally possess a much higher noise floor and lower sensitivity. Thus, the use of micro-coil in our design contributes to the forming of relatively weak but adequate magnetic field and thereby a better noise performance. Our future works will focus on the fabrication and experimental analysis of the prototype.

## Figures and Tables

**Table 1:** Structural dimensions of proposed Micro-electromechanical System (MEMS) gyroscope.

Symbol	Value	Unit	Symbol	Value	Unit	Symbol	Value	Unit
*l*_1_	1200	µm	*w*_5_	60	µm	*w*_13_	15	µm
*l*_2_	1430	µm	*w*_6_	10	µm	*w*_14_	5	µm
*l*_3_	380	µm	*w*_7_	10	µm	*w*_15_	1	µm
*l*_4_	327	µm	*w*_8_	80	µm	*w*_16_	60	µm
*w*_1_	42	µm	*w*_9_	45	µm	*a*	15	µm
*w*_2_	80	µm	*w*_10_	40	µm	*b*	4	µm
*w*_3_	80	µm	*w*_11_	30	µm	*l*	10	µm
*w*_4_	105	µm	*w*_12_	20	µm	*d*_0_	4	µm

**Table 2:** The side length of each single loop of the multi-turn coil.

Symbol	Value	Unit	Symbol	Value	Unit
*L*_1_	90	µm	*L*_5_	450	µm
*L*_2_	180	µm	*L*_6_	540	µm
*L*_3_	270	µm	*L*_7_	630	µm
*L*_4_	360	µm	*L*_8_	720	µm

**Table 3:** Extremums of selected points in sensitivity curves.

Gap (µm)	Extremum (mT/µm)	Gap (µm)	Extremum (mT/µm)
60	0.002069	120	0.0002069
80	0.001433	140	0.0006486
100	0.001066	160	0.0005198

**Table 4:** The inherent frequencies of first four modes.

Mode	Frequency (Hz)
1	5482.4
2	5557.3
3	5568.1
4	6092.1

**Table 5:** Partial design values of proposed micro-gyroscope.

Parameter	Value	Unit
Sense-mode effective mass (*m_s_*)	1.44 × 10^−6^	Kg
Comb thickness (*h*)	100	µm
Comb gap (*e*)	4	µm
Number of tuning combs (*n*)	2000	
Vacuum permittivity (ε)	8.854 × 10^−12^	F/m
Offset of Drive voltage (*V_d_*)	10	V
Amplitude of Drive voltage (*V_a_*)	5	V
